# Cerulenin inhibits unsaturated fatty acids synthesis in *Bacillus subtilis* by modifying the input signal of DesK thermosensor

**DOI:** 10.1002/mbo3.154

**Published:** 2014-02-14

**Authors:** Lucía Porrini, Larisa E Cybulski, Silvia G Altabe, María C Mansilla, Diego de Mendoza

**Affiliations:** 1 Instituto de Biología Molecular y Celular de Rosario (IBR), Consejo Nacional de Investigaciones Científicas y TécnicasRosario, Argentina; 2 Departamento de Microbiología Facultad de Ciencias Bioquímicas y Farmacéuticas, Universidad Nacional de Rosario. Ocampo y EsmeraldaRosario, Argentina

**Keywords:** *Bacillus subtilis*, cerulenin, thermosensor, unsaturated fatty acids

## Abstract

*Bacillus subtilis* responds to a sudden decrease in temperature by transiently inducing the expression of the *des* gene encoding for a lipid desaturase, Δ5-Des, which introduces a double bond into the acyl chain of preexisting membrane phospholipids. This Δ5-Des-mediated membrane remodeling is controlled by the cold-sensor DesK. After cooling, DesK activates the response regulator DesR, which induces transcription of *des*. We show that inhibition of fatty acid synthesis by the addition of cerulenin, a potent and specific inhibitor of the type II fatty acid synthase, results in increased levels of short-chain fatty acids (FA) in membrane phospholipids that lead to inhibition of the transmembrane-input thermal control of DesK. Furthermore, reduction of phospholipid synthesis by conditional inactivation of the PlsC acyltransferase causes significantly elevated incorporation of long-chain FA and constitutive upregulation of the *des* gene. Thus, we provide in vivo evidence that the thickness of the hydrophobic core of the lipid bilayer serves as one of the stimulus sensed by the membrane spanning region of DesK.

## Introduction

When bacteria are exposed to temperatures below those of their normal growth conditions, the lipids of their membrane become rigidified, leading to a suboptimal functioning of cellular activities (Mansilla and de Mendoza 2005; Mansilla et al. 2008). These organisms can acclimate to such new conditions decreasing the transition temperature of their membrane lipids, this is, the temperature at which membrane lipid bilayers undergo a reversible change of state from a liquid-crystalline (disordered) to a gel (ordered) array of the fatty acyl chains. In most bacteria, the role of introducing acyl chain disorder is fulfilled by unsaturated fatty acids (UFA), which have much lower transition temperatures than saturated fatty acids (Cronan and Gelmann 1973). Desaturation of the acyl chains of membrane phospholipids results in an increase in the membrane lipid bilayer fluidity, with restoration of normal cell function at the lower temperature.

Cold shock imposes severe constraints on the biophysical properties of *Bacillus subtilis* cytoplasmic membrane (Mansilla et al. 2004). In laboratory settings, a sudden temperature downshift, from 37 to 25 or 20°C, is used to trigger in *B. subtilis* a transiently transcriptional induction of the *des* gene coding for their sole lipid desaturase, Δ5-Des. This enzyme introduces double bonds in Δ5 positions of the acyl chain of preexisting membrane phospholipids (Aguilar et al. 1998; Altabe et al. 2003). This short-term membrane adaptation requires a canonical two-component regulatory system comprising the histidine kinase DesK and the response regulator DesR (Aguilar et al. 2001) (see Fig. S1). Upon cooling, DesK phosphorylates DesR, which stimulates the expression of Δ5-Des (Albanesi et al. 2004; Cybulski et al. 2004). By introducing a double bond into saturated lipids, Δ5-Des induces a kink in the fatty acids (FA) that increases membrane disorder, offsetting the fluidity decrease that otherwise accompanies cooling. This DesK-dependent desaturation of membrane phospholipids enhances survival of *B. subtilis* at low temperatures (Weber et al. 2001). Although the structure of full-length DesK has not yet been solved, structural studies of the catalytic core of DesK highlights the plasticity of the central Dimerization and Histidine phosphotransfer domain and suggest an important role of the transmembrane (TM) sensor domain in catalysis regulation, either by modifying the mobility of the ATP-binding domains for autokinase activity or by modulating binding of DesR to sustain the phosphotransferase and phosphatase activity (Albanesi et al. 2009). A model in which the TM domain of DesK promotes these structural changes through conformational signals transmitted by the membrane-connecting two-helical coiled-coil was postulated (Albanesi et al. 2009).

DesK is a multipass TM sensor and its activation upon a decrease in the ambient temperature appears intimately related to a decrease in the order of the acyl chain of membrane phospholipids (Cybulski et al. 2002). However, the mechanism that allows DesK to discriminate the lipid environment to promote membrane remodeling upon a drop in environmental temperature remains fragmentarily understood. Reconstitution of full-length DesK into proteoliposomes showed that, whereas the structure of the lipid head group does not affect thermosensing, the length of the acyl chains, that determine the thickness of the hydrophobic core of the lipid bilayer, exerts a profound regulatory effect on kinase domain activation at low temperatures (Martín and de Mendoza 2013). Thus, a likely hypothesis is that at low temperature, the membrane becomes thicker due to an increase in the lipid order and this change in bilayer thickness could be sensed by the TM surface of DesK, favoring its autokinase activity. However, this hypothesis is challenged by the fact that the reconstitution experiments were performed in phosphatidylcholine (PC) vesicles containing straight-chain monounsaturated FA of different chain length (Martín and de Mendoza 2013). Nevertheless, PC is absent in *B. subtilis*, which instead contains phosphatidylethanolamine and phosphatidylglycerol with acyl chains mainly composed of branched-chain FA.

**Figure 1:**
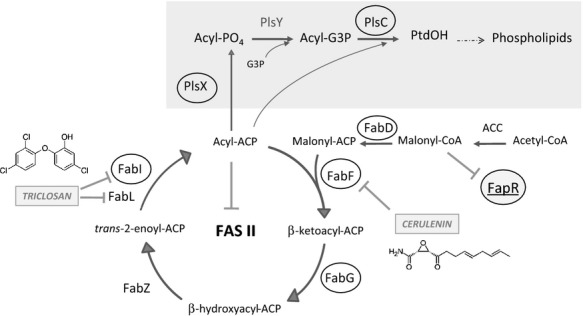
Pathway of lipid synthesis in *Bacillus subtilis*. Elongation of fatty acids (FA) is catalyzed by the type II fatty acid synthase (FASII) via a repeated cycle of condensation, reduction, dehydration, and a second reduction of carbon–carbon bonds, giving rise to acyl-acyl carrier protein (acyl-ACP) with two additional methylene groups at the end of each cycle. Generation of malonyl-CoA by acetyl-CoA carboxylase (ACC) is required to start the cycle of chain elongation by the complex. Phospholipid synthesis (shaded) initiates by the action of PlsX which converts acyl-ACPs to acyl-PO_4_. Then PlsY transfers the acyl moiety to the 1 position of glycerol-3-P (G3P) to form acyl-G3P. Acylation of the two position to form phosphatidic acid (PtdOH) is catalyzed by PlsC. Fungal toxin cerulenin inhibits the elongation condensing enzyme FabF, precluding not only FA but also phospholipid synthesis. The last step of the elongation cycle, catalyzed by enoyl-ACP reductases (FabI and FabL) is inhibited by triclosan. Expression of the genes coding for enzymes surrounded by ellipses is repressed by FapR, whose activity is, in turn, antagonized by malonyl-CoA.

In this paper, we have investigated the mechanism by which the antibiotic cerulenin, a specific inhibitor of FA synthesis (Fig. 1), abolishes the cold-induced UFA production. We found that the key change lies in production of an excess of FA of short chain length which are incorporated into membrane phospholipids. This is sufficient to alter the lipid–protein interaction required for activation of DesK at low temperature. Furthermore, artificially increasing the synthesis and incorporation of long-chain FA into *B. subtilis* membranes results in constitutive expression of *des* at high temperature. Our results strongly suggest that the thickness of the bilayer is an important parameter regulating the signaling state of DesK associated to its native plasma membrane. These findings accord with previous in vitro studies aimed at understanding how the compositional and functional diversity of the surrounding membrane modulates DesK sensor function.

## Materials and Methods

### Bacterial strains and growth conditions

Bacterial strains and plasmids used in the present study are listed in Table 1. *Escherichia coli* and *B. subtilis* strains were routinely grown in Luria Bertani (LB) broth at 37°C (Sambrook et al. 1989). Spizizen salts (Spizizen 1958), supplemented with 0.5% glucose, 0.01% each tryptophan and phenylalanine, and trace elements (Harwood and Cuttings 1990) were used as the minimal medium for *B. subtilis*. This medium was designated MM. Antibiotics were added to media at the following concentrations: erythromycin (Erm) 0.5 *μ*g mL^−1^; lincomycin (Lm) 12.5 *μ*g mL^−1^; chloramphenicol (Cm) 5 *μ*g mL^−1^; kanamycin (Km) 5 *μ*g mL^−1^; ampicilin (Amp): 100 *μ*g mL^−1^, spectinomycin (Sp): 100 *μ*g mL^−1^. For the experiments involving *desKC* and *plsC* expression under the control of the inducible promoters P*xylA* and P*spac*, 0.01% xylose and 1 mmol/L Isopropyl β-D-1-thiogalactopyranoside (IPTG) were added, respectively.

**Table 1:** Bacterial strains and plasmids.

Strain or plasmid	Relevant characteristics^1^	Source or reference
*Bacillus subtilis* strains		
JH642	*trpC2 pheA1*	Laboratory stock
AKP3	JH642 *amyE*::P*des*–*lacZ*; Cm^r^	Aguilar et al. (2001)
CM21	AKP3 *desKR*::Km, *thrC*::P*xylA-desR*; Km^r^ Sp^r^ Cm^r^	Albanesi et al. (2004)
GS77	JH642 *fabF1* (FabFI108F) cerulenin-resistant	Schujman et al. (1998)
BLUP87	GS77 *amyE*::P*des*–*lacZ;* Cm^r^	This study
BLUP34	JH642 *plsC*::P*spacOid-plsC;* Cm^r^	This study
BLUP102	JH642 *plsC*::P*spacOid-plsC;* Sp^r^	This study
BLUP103	BLUP102 *amyE*::P*des-lacZ;* Cm^r^ Sp^r^	This study
*Escherichia coli* strain		
DH5*α*	*supE44 thi-1* Δ*lacU169* (Φ80*lacZ*ΔM15) *endA1 recA1hsdR17gyrA96 relA1trp-6 cysT329*::*lac inm*^*pI(*209)^	Laboratory stock
Plasmids		
pCR-BluntII-TOPO	pCR-Blunt II-TOPO *E. coli* cloning vector; Km^r^	Invitrogen
pJM116	Integrative vector to construct transcriptional fusions to *lacZ*; integrates at the *amyE* locus of *B. subtilis*; Cm^r^	Perego (1993)
pHPKS	*B. subtilis* replicative vector of low copy number, Erm^r^ Lm^r^	Johansson and Hederstedt (1999)
pCm::Sp	Antibiotic switching vector; Amp^r^, Sp^r^	Steinmetz and Richter (1994)
pDH88	Integrative plasmid containing the IPTG-inducibleP*spac* promoter; Cm^r^	Henner (1990)
pMUTIN4	Integrative plasmid containing the IPTG-inducible P*spac-oid* promoter; Erm^r^ Lm^r^	Vagner et al. (1998)
pAR11	Contains P*des* cloned into the *Eco*RI–*Bam*HI sites of pJM116	Aguilar et al. (2001)
pCM9	P*xylA*-*desKC* cloned into pHPKS	Albanesi et al. (2004)
pLUP30	P*spacOid* of pMUTIN4 cloned into the *Eco*RI–*Hind*III sites of pDH88	This study
pLUP32	5´ end of the *plsC* gene cloned into the *Hind*III–*Sph*I sites of pLUP30	This study
pLUP124	P*xylA*-*desK* cloned into pHPKS	This study

1 Cm^r^, Sp^r^, Km^r^, Erm^r^, Lm^r^, and Amp^r^ denote resistance to chloramphenicol, spectinomycin, kanamycin, erythromycin, lincomycin, and ampicillin, respectively.

### Genetic techniques

*Escherichia coli* competent cells were transformed with supercoiled plasmid DNA by the calcium chloride procedure (Sambrook et al. 1989). Transformation of *B. subtilis* was carried out by the method of Dubnau and Davidoff-Abelson (1971). The *amy*^*−*^ phenotype was assayed with colonies grown during 48 h in LB starch plates, by flooding the plates with 1% I_2_-KI solution (Sekiguchi et al. 1975). *amy*^+^ colonies produced a clear halo, while *amy*^*−*^ colonies gave no halo.

### Plasmid and strains construction

In all cases, DNA fragments were obtained by PCR using the oligonucleotides described in the text (restriction sites underlined). Chromosomal DNA from strain JH642 was used as the template. The PCR products of the expected sizes were cloned into pCR-Blunt II-Topo (Promega, Madison, WI) and transformed in *E. coli* DH5*α* (Sambrook et al. 1989). Plasmid DNA was prepared using the Wizard DNA purification system (Promega Life Science) and sequenced to corroborate the identity and correct sequence of the cloned fragments.

To generate an integrative vector containing a tightly regulated IPTG-inducible P*spac-oid* promoter, but without a transcriptional fusion to *lacZ*, we decided to replace the P*spac* promoter of plasmid pDH88 (Henner 1990) by P*spac-oid* of pMUTIN4 (Vagner et al. 1998). A 603-bp DNA fragment, generated by PCR using primers TerPspacOid Up (CGTGAGGAATTCAATAAAACGAAAGGCTCAGTCGAAAGA) and TerPspacOid Lw (CTGGGATCCGCATGCTGTACATCAAGCTTAATTGTGAG), containing P*spac-oid* promoter from pMUTIN4, was digested with *Eco*RI and *Hind*III and cloned into the integrative plasmid pDH88, previously digested with the same enzymes to release its P*spac*, yielding plasmid pLUP30.

The *plsC* isogenic conditional mutant was constructed as follows: a 489 bp DNA fragment, corresponding to the ribosome binding site and a 5′ portion of *plsC* gene, was obtained by PCR amplification using the oligonucleotides PyhdOHindIII (AATCAAAGCTTACGACAAAGGAAGTGCGAT) and YhdOSphI (TTTTTTGCATGCTTCTTTTCCGCTTGAA). The fragment was cloned into the *Hind*III and *Sph*I sites of vector pLUP30, which allows expression of the *plsC* gene under the control of P*spac-oid*. The resulting plasmid was named pLUP32. This construct was then integrated by a single-crossover event at the *plsC* locus of *B. subtilis* JH642, yielding strain BLUP34. This approach results in the conditional inactivation of *plsC* gene, whose expression can be controlled by P*spac-oid*. This strain was checked by PCR to ensure that the plasmid is integrated in the correct site.

To allow the introduction of the reporter fusion contained in the plasmid pJM116 (Cm^r^) (Perego 1993), the chloramphenicol cassette present in the *plsC* locus of BLUP34 was changed for a spectinomycin resistance cassette through transformation and homologous recombination using the plasmid pCm::Spc (Steinmetz and Richter 1994). The resulting strain was named BLUP102. To introduce the transcriptional fusion of *lacZ* to the promoter region of the desaturase gene (P*des-lacZ*) into the *plsC* conditional mutant, the plasmid pAR11 (Aguilar et al. 2001) was linearized with *Sca*I and introduced by a double crossover event at the *amyE* locus of BLUP102 chromosome yielding strain BLUP103.

To ectopically express the P*des-lacZ* fusion in a *B. subtilis* JH642 cerulenin-resistant strain, plasmid pAR11 (Aguilar et al. 2001) was linearized with *Sca*I and introduced by a double crossover event at the *amyE* locus of strain GS77 (Schujman et al. 1998) giving rise to strain BLUP87.

To construct plasmid pLUP124, a 1227 bp DNA fragment containing *desK* was obtained by PCR amplification using the oligonucleotides DesKB33-Up (AGTAACATGGATCCCAGAAAATGAGGTAAGATC) and desKP-Dw (GCTGATCTTCTGCAGTAAATATACTAATC). The fragment was cloned into the *Bam*HI and *Pst*I sites of vector pARD7 (pHPKS replicative vector containing the P*xyl* promoter; M. C. Mansilla, pers. comm.).

### *β*-galactosidase assays

*Bacillus subtilis* cells harboring a P*des–lacZ* chromosomal fusion were grown in MM at 37°C to an OD_525_ of 0.35, then were split and half of the culture was treated with either 2.5 *μ*g mL^−1^ of cerulenin (MIC 5 *μ*g mL^−1^, Schujman et al. 2001), 0.4 *μ*g mL^−1^ of triclosan (MIC 2 *μ*g mL^−1^, Heath et al. 2000), or 0.01% xylose as indicated in each experiment. Then cultures were transferred to 25°C or 37°C, as indicated. *B. subtillis* BLUP103 cells were grown ON in MM at 37°C supplemented with 0.2 mmol/L IPTG to allow the expression of *plsC*. Cells were washed twice, resuspended in MM to OD_525_ values of 0.03 in the presence or absence of 1 mmol/L IPTG and incubated at 37°C to an OD_525_ of 0.35, then were split and incubated at 25 or 37°C. After each treatment, samples were taken at 1-h intervals and assayed for *β*-galactosidase activity as described previously (Mansilla and de Mendoza 1997). The specific activity was expressed in Miller units (Miller 1972). The results shown are the average of three independent experiments.

### FA analyses

For the measurement of UFA biosynthesis, cultures of strains AKP3 (wild-type) and BLUP87 (cer^r^) were grown in MM at 37°C to an OD_525_ of 0.25 and then half of this culture was supplemented with 2.5 *μ*g mL^−1^ of cerulenin for 45 min. When the strains reached an OD_525_ of 0.35, 2 mL of these cultures were labeled with 0.2 μCi of [^14^C]palmitate (specific activity, 58 mCi/mmol/L) and further shifted to 25°C for 5 h. Following incubation, cells were collected and lipids were prepared according to the method of Bligh and Dyer (1959). The FA methyl esters were prepared by transesterification of glycerolipids with 0.5 mol/L sodium methoxide in methanol (Christie 1989) and separated into saturated FA and UFA fractions by chromatography on 10% silver nitrate-impregnated Silica Gel G plates (0.5-mm thickness; Analtech Inc., Newark, DE). About 11,000 cpm of radioactivity were loaded into each lane. Chromatographic separation was achieved in a toluene solvent system at −20°C and detected by using a Typhoon 9200 PhosphorImager screen (STORM840; GE Healthcare Argentina S.A., Buenos Aires, Argentina). The radioactivity levels of the spots were quantified by ImageQuant 5.2 (GE Health Care Argentina S.A.).

### Analysis of FA by GC-MS

To determine the FA composition, AKP3 cells were grown in MM to an OD_525_ of 0.35 at 37°C. Cultures were treated or not with 2.5 *μ*g mL^−1^ cerulenin and then shifted to 25°C for 5 h. Total lipids were extracted and transesterified to yield FA methylesters as described above. The FA methylesters were analyzed in a Perkin–Elmer Turbo Mass gas chromatography–mass spectrometer on a capillary column (30 mm by 0.25 mm in diameter Varian) of 100% dimethylpolysiloxane (PE-1; Perkin-Elmer, Waltham, MA). Helium at 1 mL/min was used as the carrier gas, and the column temperature was programmed to rise by 4°C/min from 100 to 320°C. Branched-chain FA, straight-chain FA, and UFA used as reference compounds were obtained from Sigma Chemical Co (St. Louis, MO).

*Bacillus subtilis* strain BLUP103 was grown overnight at 37°C in MM supplemented with 0.2 mmol/L IPTG. On the following day, fresh cultures were started by washing twice, resuspended in MM and grown at 37°C in the presence or absence of 1 mmol/L IPTG. After 6 h of growth in the absence of IPTG addition, cells stopped growing because of PlsC depletion. At this point, 50 mL samples cultures were collected and lipids were prepared and analyzed as described above.

## Results

### Cerulenin inhibits des transcription and UFA synthesis at low temperatures

During previous work aimed to test whether preexistent lipids were able to regulate the expression of Δ5-Des (Cybulski et al. 2002; and S. G. Altabe, pers. comm.), we found that cerulenin, a specific inhibitor of FabF (Fig. 1), the sole condensing enzyme accomplishing acyl chain elongation in *B. subtilis* (Schujman et al. 2001) repressed the induction of the *des* gene during cold shock. This was an unexpected observation as previous results of our laboratory showed that inhibition of FA synthesis by cerulenin significantly increases transcription of at least 10 genes, contained in five different operons, encoding key enzymes involved in FA and phospholipid biosynthetic pathways (Schujman et al. 2001, 2003).

**Figure 2:**
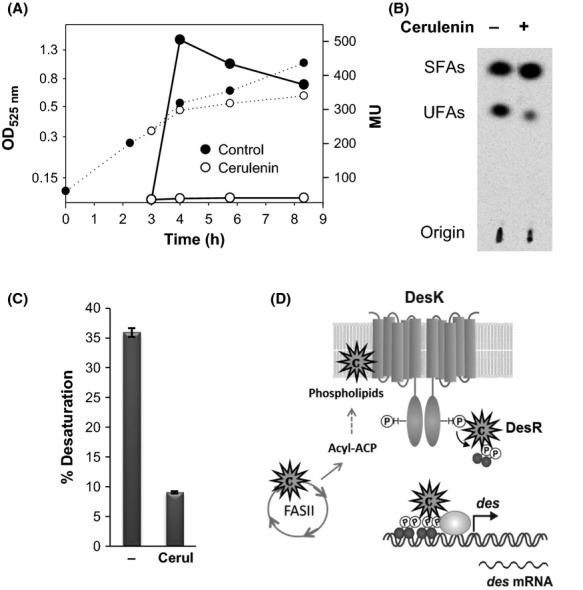
Effect of cerulenin on expression of *des* promoter and autoradiogram of the products of [^14^C]palmitate labeling. (A) *Bacillus subtillis*AKP3 cells (*amyE:*:P*des-lacZ,* cer^S^) were grown in MM at 37°C to an OD_525_ of 0.35 and then cultures were split in two. One half was supplemented with cerulenin 2.5 *μ*g mL^−1^ (white circles), while the other remained untreated (black circles), then cultures were transferred to 25°C. *β*-galactosidase-specific activities (in Miller units, MU) were determined at the indicated time intervals. Dotted lines: OD_525_; solid lines: *β*-galactosidase-specific activities. (B) UFAs synthesized by strain AKP3 after a temperature downshift, in the presence or the absence of cerulenin. Cultures were labeled with 0.2 μCi of [^14^C]palmitate and incubated at 25°C for 5 h, as described in Material and Methods. Lipids were extracted and transesterified; the resulting methyl esters were separated into saturated (SFAs) and unsaturated (UFAs) fractions by chromatography on silver nitrate-impregnated silica plates. About 11,000 cpm of radioactivity was loaded into all lanes. (C) Percentages of UFA synthesis. The radioactivity levels of the spots of the UFA and SFA shown in panel B were quantified by ImageQuant 5.2. Results are expressed as percentages of the total methyl esters recovered. Values are the average of three independent experiments. (D) Cerulenin could block different steps in the transmission of the cold signal. The putative sites of action of cerulenin are indicated as asterisks.

As this intriguing preliminary observation could indicate a new level of control in the well-studied Des signal transduction pathway, we decided to examine in detail the mechanism of *des* promoter regulation by cerulenin. We first assayed the effect of cerulenin on expression of the *des* gene using strain AKP3 (Aguilar et al. 2001). This strain contains the *lacZ* reporter gene under the control of the *des* promoter, integrated ectopically at the nonessential *amyE* locus. AKP3 was grown until early exponential phase at 37°C and then transferred to 25°C. Half of the culture was treated with sublethal concentration of cerulenin (2.5 *μ*g mL^−1^). As shown in Figure 2A, the *β*-galactosidase levels of AKP3 cells growing with cerulenin were five times lower than the untreated cultures. Consistently, after a temperature downshift, AKP3 cells treated with cerulenin synthesized lower levels of UFAs than cells growing in the absence of the antibiotic (Fig. 2B and C). These data showed that cerulenin strongly inhibit *des* gene induction at low temperature.

### The TM segments of DesK are essential for cerulenin repression of des transcription

**Figure 3:**
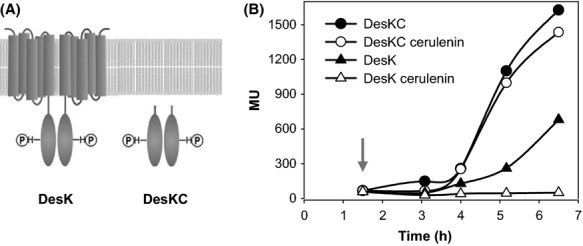
The TM domain of DesK is required for cerulenin repression of *des* transcription. (A) Scheme of DesK and DesKC proteins. (B) Cells of *Bacillus subtillis* CM21 (*desKR::*Km *thr*::P*xyl-desR, amyE::*P*des-lacZ*) transformed with plasmid pCM9 (P*xyl-desKC*) or pLUP124 (P*xyl-desK*) were grown in LB medium at 37°C to an OD_525_ of 0.3. Cultures were supplemented with 0.01% of xylose and then were treated with cerulenin 2.5 *μ*g mL^−1^ (white circles) or untreated (black circles). Cultures were further transferred to 25°C. *β*-galactosidase specific activities (in Miller units, MU) were determined at the indicated time intervals. Addition of the supplements is indicated by the arrow. Values are representative of three independent experiments.

Repression of *des* transcription by cerulenin could be caused by alternative mechanisms that would block different steps in the transmission of the cold signal (Fig. 2D). It follows that, by a direct action or through inhibition of de novo lipid synthesis, cerulenin could affect sensing properties of DesK, its autophosphorylation and/or the flux of phosphate from DesK-P to the DesR transcription factor. Alternatively, cerulenin could promote dissociation of DesR-P from the *des* promoter. To distinguish between these possibilities, we used strain CM21, which carries a DesK null mutation, expresses *desR* from the xylose-inducible P*xyl* promoter and contains a P*des*–*lacZ* fusion integrated at the *amyE* locus (Albanesi et al. 2004). This strain was transformed either with plasmid pCM9 expressing an N-terminal truncated form of DesK, named DesKC, which lacks the complete TM region, but retains the catalytic core of DesK (Fig. 3A) or with plasmid pLUP124, which express full-length DesK. It has been previously shown that when DesKC is expressed in strain CM21 the P*des*–*lacZ* fusion is constitutively expressed, even at 37°C, and is not influenced by the addition of exogenous UFAs. This behavior probably takes place because DesKC, which is unable to respond to membrane signals, remains locked in a kinase-dominant state (Albanesi et al. 2004). As shown in Figure 3B, when CM21 is complemented with wild-type DesK cerulenin addition represses *des* transcription, conversely *β*-galactosidase activity of CM21/pCM9 was not repressed by cerulenin. These results indicate that the antibiotic does not inhibit the autokinase or the phosphotransferase activities of the truncated DesKC protein, or the activation of the *des* promoter by phosphorylated DesR. These data support the notion that the TM domain of DesK is essential to sense the inhibitory effect of cerulenin on *des* transcription. As it is well established that the TM domain of DesK discriminates the surrounding lipid environment to adjust the signaling state of the sensor kinase (Mansilla and de Mendoza 2005), possible changes in membrane lipid composition induced by cerulenin treatment could be responsible for shutting off the cold-induced DesK autokinase activity.

### Downregulation of des expression is linked to inhibition of FA synthesis

**Figure 4:**
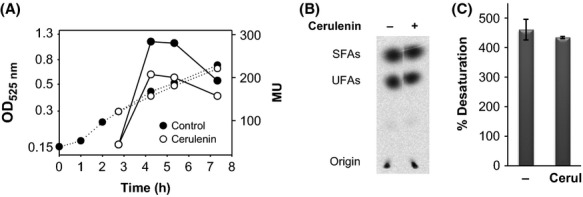
*des* expression and autoradiogram of the products of [^14^C]palmitate labeling of a cerulenin-resistant strain. (A) Cells of *B. subtillis*BLUP87 (*fabF1* cer^r^, *amyE:*:P*des-lacZ*) were grown in MM at 37°C to an OD_525_ of 0.35 and then were treated with cerulenin 2.5 *μ*g mL^−1^ (white circles) or untreated (black circles). Cultures were further transferred to 25°C. *β*-galactosidase specific activities (in Miller units, MU) were determined at the indicated time intervals. Dotted lines: OD_525,_ solid lines: *β*-galactosidase specific activities. Values are representative of three independent experiments. (B) UFAs synthesized by strain BLUP87 after a temperature downshift, in the presence or the absence of cerulenin. Cultures were challenged with [^14^C]palmitate as described in Figure 2B. About 11,000 cpm of radioactivity was loaded into all lanes. (C) Percentages of UFA synthesis. The radioactivity levels of the spots of the UFA and SFA shown in panel B were quantified by ImageQuant 5.2. Results are expressed as percentages of the total methyl esters recovered. Values are the average of three independent experiments.

It should be noted that at this stage of the work, we were unable to distinguish if inhibition of transcription of the *des* gene mediated by cerulenin was due to inhibition of its target enzyme, FabF, or by a side effect of the antibiotic, such as its insertion into the membrane. To answer this question, *des* transcription was evaluated in BLUP87, a cerulenin-resistant mutant of *B. subtilis* that contains a P*des*-*lacZ* transcriptional fusion in the *amyE* locus. Resistance to cerulenin in this strain is given by the I108F substitution of FabF (*fabF1* allele), which introduces a residue in the hydrophobic acyl chain-binding pocket that hampers the optimum interaction between the enzyme and the acyl chain of cerulenin (Schujman et al. 2001, 2008). As shown in Figure 4A, after a cold shock, cerulenin produces a slight inhibition of *des* transcription in this strain (see Fig. 2A). Moreover, the levels of UFAs synthesized by BLUP87 at low temperatures were not diminished by addition of cerulenin (Fig. 4B and C). These results rule out that cerulenin itself could modify the membrane microenvironment of DesK, leading to downregulation of *des* expression. Thus, this result strongly suggests that repression of *des* transcription is due to specific inhibition of FA synthesis by cerulenin. To confirm this idea, we tested *des* expression in the presence of triclosan, an antibiotic that inhibits other enzymes of the fatty acid biosynthetic pathway, FabI and FabL, which catalyze the NADPH reduction of enoyl-ACP to acyl-ACP during the elongation cycle of FA synthesis (Fig. 1, McMurray et al. 1998; Heath et al. 1998, 2000). Addition of sublethal concentration of triclosan (0.4 *μ*g mL^−1^) to AKP3 (cer^S^) or BLUP87 (cer^R^) cells repressed *des* transcription in both strains (Fig. S2). These results demonstrate that downregulation of *des* expression is correlated with the specific inhibition of FA synthesis and suggest that this response would be observed when any step in the pathway is blocked.

### Changes in membrane FA composition in response to cerulenin

The FA composition of strain AKP3, treated or not with cerulenin after a temperature downshift, was determined by gas chromatography–mass spectrometry (GC-MS). In agreement with the experiments shown in Figure 2B, we observed that the proportion of UFAs in cells treated with the antibiotic decreased about threefold (from 10.0 to 3.4%, Table 2). Moreover, and surprisingly, we found that the addition of cerulenin increases the production of shorter FA, as the ratio of short-chain FA (FA of chain length of C13-C15) to long-chain FA (FA of chain length C16 or longer) rose about twofold (1.1–2.2, Table 2). Finally, cerulenin seems not to affect the amount of straight-chain FA nor the iso/anteiso-branched chain FA ratio. These results strongly suggest that inhibition of *des* expression by cerulenin is due to shortening of the acyl chains of membrane lipids that would be sensed by the TM domain of DesK, acquiring a phosphatase-dominant state.

**Table 2:** Fatty acid composition of the phospholipids of strain AKP3.

	Percentage of fatty acid-type^1^		
Fatty acid	No addition	Cerulenin	±^2^
Iso C_13:0_	0.08	0.89	11.1
Anteiso C_13:0_	0.05	1.34	26.8
Iso-C_14:0_	3.47	4.49	1.3
n-C_14:0_	0.43	1.27	3.0
Iso-C_15:0_	17.90	20.36	1.2
Anteiso C_15:0_	29.29	37.50	1.3
n-C_15:0_	1.46	1.68	1.2
Iso-C_16:1_	2.36	0.75	0.3
Iso-C_16:0_	9.11	6.30	0.7
n-C_16:1_	4.40	1.76	0.4
n-C_16:0_	8.58	7.95	0.9
Iso-C_17:1_	2.33	0.70	0.3
Anteiso C_17:1_	0.93	0.20	0.2
n-C_17:0_	0.66	0.44	0.7
n-C_18:1_	0.60	1.33	2.2
UFAs	10.00	3.40	0.3
SCFA/LCFA^3^	1.1	2.2	2.0
Iso/anteiso^4^	1.1	0.9	0.8
Straight-chain FA	16.1	14.4	0.9
Iso + straight/anteiso^5^	1.5	1.2	0.8

1 Cells were grown in MM at 37°C to an OD_525_ of 0.35. Cultures were split and cerulenin (2.5 *μ*g mL^−1^) was added to one half. Cultures were further transferred to 25°C and cells harvested after 6 h of growth. Total lipids were extracted, transesterified to yield FA methylesters and subjected to GC-MS analysis. Values are representative of three experiments.

2 Ratio of percentages of antibiotic-treated cells compared to untreated cells.

3 Ratio of short-chain FA (SCFA, C13 to C15) to long-chain FA (LCFA, C16 or longer).

4 Ratio of iso-branched-chain FA to anteiso-branched-chain FA.

5 Ratio of (iso-branched-chain FA + straight-chain FA) to anteiso-branched-chain FA.

### Effect of long-chain FA on des transcription

As transcriptional repression of *des* by cerulenin seems to be a consequence of the shortening of the membrane FA chains, it is conceivable that the opposite effect can be obtained in cells whose membranes are enriched in long-chain FA. If this is the case, cells overproducing long-chain FA, could activate *des* transcription even at 37°C. It has been described that the blockade of phospholipid synthesis in *B. subtilis* by depletion of PlsC, the enzyme that acylates acyl-glycerol phosphate, leads to a very significant accumulation of free FA (Paoletti et al. 2007). Nevertheless, the accumulation of the 1-acyl-glycerol-3-P intermediate was not observed (Paoletti et al. 2007). Thus, we used strain BLUP103, which contains the *plsC* gene under the control of the IPTG-inducible P*spac* promoter and a P*des*–*lacZ* fusion integrated at the *amyE* locus. In this strain, removal of the inducer does not result in the immediate inactivation of the protein or cessation of cell proliferation, but rather, cell growth continues until the preexisting protein is diluted out by subsequent cell divisions, while the synthesis of FA continues at a very significant rate. Thus, during the transition from log phase to growth stasis of BLUP103, abnormally long-chain FA should be incorporated into membrane lipids. To test our hypothesis, we determined the FA composition of phospholipids of BLUP103 cells, grown at 25 or 37°C in the presence or in the absence of the inducer. To this end, the extracted FA was subjected to transesterification with sodium methoxide. This procedure allows analyzing by GC-MS only membrane FA esterified to glycerol. Thus, the FA percentages mostly reflect the composition of complex lipids rather than the content of free FA.

**Figure 5:**
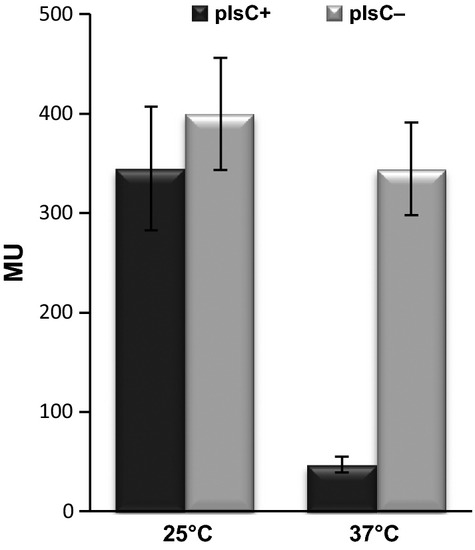
Effect of *plsC* depletion on *des* transcription. BLUP103 (P*spac-plsC amyE*::*Pdes-lacZ*) cells grown ON at 37°C in Spizizen minimal medium supplemented with 0.2 mmol/L IPTG were washed and used to inoculate fresh media, in the presence (black bars) or absence (gray bars) of 1 mmol/L IPTG. At OD_525_ of 0.35 cultures were split and incubated at 25 or 37°C. *β*-galactosidase specific activities were determined 4 h after the temperature shift. Values expressed in Miller units (MU) are representative of three independent experiments.

As expected, the mass spectrum of PlsC-depleted cells displayed a significant increase (5.6-fold) in the proportion of long-chain FA (Table 3), including the synthesis of FA of a chain length of 19–22°C. This effect was more pronounced at 37°C. In addition, depletion of PlsC at 37°C led to a threefold increase in the amount of straight-chain FA when compared with cells grown with the inducer (45.9 vs. 13.1%). To determine whether these changes in membrane lipid composition affect *des* expression, we analyzed the activity of the P*des-lacZ* transcriptional fusion in BLUP103, with or without IPTG addition, on cells grown at 37°C and after a shift to 25°C. As shown in Figure 5, similar levels of *des* transcription were observed in *plsC* cells growing at 25°C in the presence or in the absence of the inducer. However, at 37°C the activity of the *des* promoter in PlsC-depleted cells reached induction levels about eightfold higher than the levels found in presence of IPTG. These data support the notion that the presence of higher amounts of long-chain FA in the membrane of *plsC*-depleted cells stabilize DesK in a constitutive kinase state.

**Table 3:** Fatty acid composition of the phospholipids of a strain deficient in PlsC.

Fatty acid	Percentage of fatty acid type^1^					
	25°C			37°C		
	PlsC^+^	PlsC^−^	±^2^	PlsC^+^	PlsC^−^	±^2^
Iso C_13:0_	0.20	0.29		0.26	0.14	
Anteiso C_13:0_	0.52	0.20		ND	ND	
Iso-C_14:0_	1.67	1.77		2.27	0.83	
n-C_14:0_	0.43	0.50		0.70	0.44	
Iso-C_15:0_	14.69	10.89		23.44	7.04	
Anteiso C_15:0_	38.18	27.33		30.84	11.08	
n-C_15:0_	1.38	1.11		1.63	0.92	
Iso-C_16:1_	1.66	1.73		ND	0.12	
Iso-C_16:0_	3.12	4.34		6.54	4.99	
n-C_16:1_	4.25	3.99		0.28	0.32	
n-C_16:0_	7.09	7.20		7.43	10.76	
Iso-C_17:1_	4.59	5.93		ND	ND	
Anteiso C_17:1_	1.28	2.84		ND	ND	
n-C_17:0_	0.67	1.24		0.74	12.88	
Iso-C_18:0_	0.07	0.77		0.62	3.09	
n-C_18:1_	1.83	3.64		2.30	15.90	
Iso-C_19:0_	ND	0.26		0.48	2.13	
Anteiso C_19:0_	ND	0.48		0.13	1.86	
n-C_19:0_	ND	0.16		ND	0.79	
Iso-C_20:0_	ND	ND		ND	1.12	
n-C_20:0_	ND	0.40		ND	3.73	
Iso-C_21:0_	ND	ND		ND	0.39	
Anteiso C_21:0_	ND	ND		ND	0.31	
n-C_21:0_	ND	ND		ND	0.13	
Iso-C_22:1_	ND	ND		ND	0.19	
Iso-C_22:0_	ND	ND		ND	0.25	
UFAs	11.8	14.5	1.2	0.3	0.6	2.0
LCFA/SCFA^3^	0.8	1.4	1.8	0.7	3.9	5.6
Iso/anteiso^4^	0.6	0.8	1.3	1.1	1.4	1.3
Straight-chain FA	15.7	18.2	1.2	13.1	45.9	3.5
Iso + straight/anteiso^5^	0.9	1.1	1.2	1.5	3.4	2.3

ND, not detected.

1 BLUP103 (P*spac-plsC*) cells grown ON at 37°C in MM supplemented with 0.2 mmol/L IPTG were washed and used to inoculate fresh media, in the presence (PlsC^+^) or absence (PlsC^−^) of 1 mmol/L IPTG. At DO_600_ of 0.35 cultures were split and incubated at 25 or 37°C for 6 h. When growth stopped because of PlsC depletion, total lipids were extracted, transesterified to yield FA methylesters and subjected to GC-MS analysis.

2 Ratio of percentages of not induced cells compared to induced cells.

3 Ratio of long-chain FA (LCFA, C16 or longer) to short-chain FA (SCFA, C13 to C15).

4 Ratio of iso-branched-chain FA to anteiso-branched-chain FA.

5 Ratio of (iso-branched-chain FA + straight-chain FA) to anteiso-branched-chain FA.

## Discussion

*Bacillus subtilis* is a typical mesophile that can be found in the upper layers of the soil, which is subjected to temperature changes both during the course of the day, and over longer time periods, as a consequence of seasonal changes. Rapid and severe temperature downshifts elicit genetic and cellular adaptive reactions that are collectively known as the cold shock stress response (Weber and Marahiel 2002). As cold shock imposes marked changes on the biophysical properties of *B. subtilis* cytoplasmic membrane (Mansilla et al. 2004), temperature sensing is important to optimize membrane fluidity in this organism. The cold signal induces transcription of the *des* gene, leading to the introduction of double bonds into the acyl chains of phospholipids. In this paper, we show that the fungal antibiotic cerulenin represses *des* induction during cold shock. As cerulenin has been extensively used as a tool to understand several aspects of lipid metabolism (Furukawa et al. 1993; Heath and Rock 1995; Loftus et al. 2000; Schujman et al. 2003), the main objective of this work was to uncover the mechanism by which this antibiotic causes inhibition of *des* transcription.

Cerulenin inhibits lipid synthesis in *B. subtilis* by the covalent active site-directed inactivation of the FabF condensing enzyme, the enzyme that catalyzes the condensation of malonyl-CoA with acyl-ACP (Fig. 1). Cessation of FA synthesis caused by cerulenin also inhibits the production of phosphatidic acid (PtdOH), the precursor of membrane phospholipids (Fig. 1). However, we have shown here that inhibition of phospholipid synthesis by PlsC depletion does not impair *des* transcription after cold shock (Fig. 5). So, inhibition of *des* expression by cerulenin is linked to inhibition of FA synthesis rather than to phospholipid synthesis. Besides, we showed that the addition of sublethal levels of cerulenin, that do not inhibit growth of *B. subtilis* wild-type strains, alters the length of the acyl chains of membrane phospholipids. In fact, we found that the membrane FA composition of *B. subtilis* treated with cerulenin is clearly biased toward shorter-chain fatty acyl groups (Table 2).

An important question raised by this work is: how could the FA chain length of *B. subtilis* phospholipids be decreased by cerulenin? FabF forms part of the *fap* regulon of *B. subtilis* (comprising almost all the proteins that catalyzes the later steps of the FASII cycle as well as the earlier steps of phospholipid synthesis, Fig. 1), which is transcriptionally regulated by the FapR repressor. Binding of the repressor to its target sequences is modulated by the levels of malonyl-CoA (Schujman et al. 2006). Antibiotics that specifically inhibit the FASII cycle augment the intracellular levels of malonyl-CoA, which in turn release FapR from its binding sites, increasing the expression of the *fap* regulon (Schujman et al. 2003). In *B. subtilis,* two genes involved in the acyl transfer step of phospholipid synthesis, *plsX* and *plsC*, are upregulated by inactivation of the FASII cycle (Schujman et al. 2003). Although the experiments shown here suggest that FabF is responsible for the decrease in the acyl chain length of FA of cells exposed to cerulenin, it should be noted that this leftacteristic is dependent upon the competition between the elongation activity of the FASII and the rate of incorporation of FA by the acyltransferase system (Yao and Rock 2013). We envision that in cerulenin-treated cultures, the combined effect of a decrease in the relative rate of the acyl chain elongation by FabF and overproduction of PlsX and PlsC, are responsible for the increased incorporation of FA of shorter chain length. This proposal agrees with the observation that cells with normal rate of FA synthesis, but decreased PlsC activity accumulates long-chain FA (Table 3).

Reconstitution of DesK into bilayers of PC containing acyl chains of different length showed that the longer the FA (the thicker the bilayer) the greater its kinase activity (Martín and de Mendoza 2013). Nevertheless, these data were obtained with vesicles made of phospholipids that are not normally present in *B. subtilis* membranes. In this paper, we demonstrate that modulation of DesK kinase activity by the thickness of the bilayer indeed take place in vivo under isothermal conditions, using *B. subtilis* native phospholipids. But, how could the acyl-chain length of membrane phospholipids influence DesK regulation? Changes in membrane thickness can alter the activity of membrane proteins by modifying the orientation or conformation of TM regions (Lee 2003; Cybulski and de Mendoza 2011). Chill stress, among other effects, causes an increase in membrane thickness generated by a decrease in the disorder of the acyl-chains of phospholipids that accompanies cooling (Rafael Oliveira, personal communication).

Periplasmic-sensing histidine kinases comprise the largest group of membrane-bound sensor kinases. They contain a significantly large extracytoplasmic input domain, which generally detects signals by direct interaction with chemically defined small molecules (Mascher et al. 2006). On the other hand, DesK belongs to a group of histidine kinases with the sensing mechanism linked to the TM regions. The molecular basis by which this group of histidine kinases sense environmental signals is largely unknown. We have recently reported that the multimembrane-spanning domain from DesK could be simplified into a chimerical single-membrane-spanning minimal sensor (MS)-DesK that fully retains in vivo and in vitro the cold-sensing properties of the parental system (Cybulski et al. 2010). Mutational and biochemical analysis of this membrane-bound chimera showed that two hydrophilic residues near the N-terminus of DesK's first TM segment are critical for its cold-activation (Cybulski et al. 2010). This region has been named the “buoy,” as its hydrophilicity drives it toward the lipid/water interface, while the hydrophobicity of surrounding residues anchors the buoy to the membrane and can potentially pull it into the membrane interior. The “sunken-buoy” model of thermosensing poses that as the membrane thickens upon cooling, the hydrophilic buoy is pulled into the hydrophobic membrane, an energetically unfavorable situation that elicits conformational changes within the DesK protein that increase the activity of its histidine kinase domain (Cybulski et al. 2010). While the precise structural changes within the TM region remain uncertain, the results described here by manipulating in vivo the chain length of *B. subtilis* phospholipids, either by inhibiting the elongation activity of FASII or the rate of incorporation by the acyl transfer system, suggest that DesK regulation is indeed linked to changes in membrane thickness that could trigger buoy-dependent conformational changes in this integral membrane cold-sensor.

## Supporting Information

Figure S1 The Des signaling pathway for regulation of UFAs synthesis in *Bacillus subtilis*. DesK could assume different signaling states in response to changes in membrane fluidity. An increase in the order of the acyl chains of membrane lipids (less fluid membrane) promotes a kinase-dominant state of DesK, which autophosphorylates and transfers the phosphate group to DesR. DesK-mediated phosphorylation of DesR enables interaction of DesR-P with *des* promoter and RNA polymerase, resulting in transcriptional activation of *des*. Then Δ5-Des is synthesized and desaturates the acyl chains of membrane phospholipids. These newly synthesized UFAs cause a decrease in the order of membrane lipids (more fluid membrane) favoring a phosphatase-dominant state of DesK, leading to dephosphorylation of DesR and thus turning off *des* transcription.

Figure S2 Effect of triclosan on *des* transcription in cerulenin-sensitive or cerulenin-resistant strains. Cells of B. *subtillis* AKP3 (*fabF* cer^S^, *amyE*::P*des-lacZ*) (A) or BLUP87 (*fabF1* cer^r^, *amyE*::P*des-lacZ*) (B) were grown in LB medium at 37°C to an OD_525_ of 0.35 and then were treated with triclosan 0.4 *µ*g mL ^−1^ (white circles) or untreated (black circles). Cultures were further transferred to 25°C. *β*-galactosidase specific activities (in Miller units, MU) were determined at the indicated time intervals. Dotted lines: OD*525*, solid lines: *β*-galactosidase specific activities. Values are representative of three independent experiments).
