# Supplementary figures and images for: Cerulenin inhibits unsaturated fatty acids synthesis in Bacillus subtilis by modifying the input signal of DesK thermosensor
Source: Microbiologyopen. 2014 Feb 14;3(2):213–24. doi: 10.1002/mbo3.154 (PMC3996569; doi:10.1002/mbo3.154)

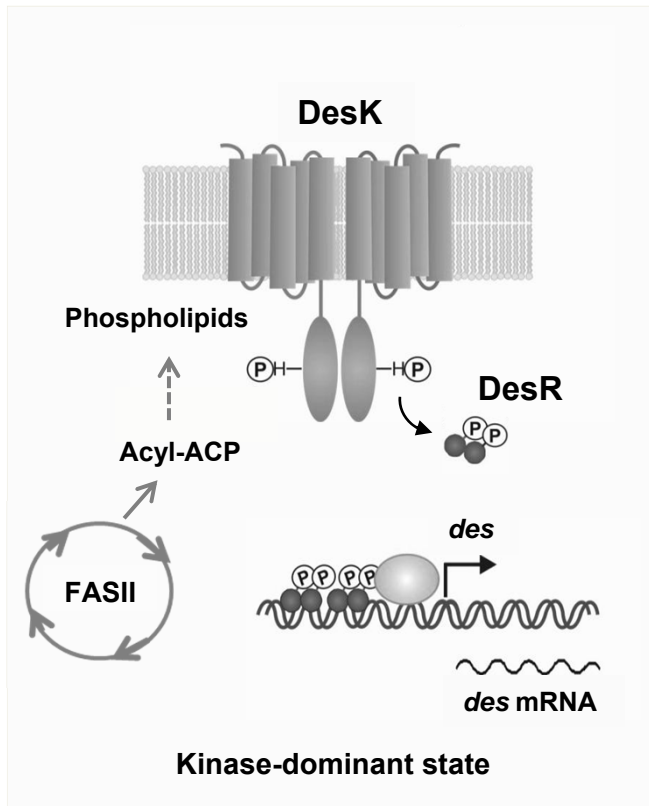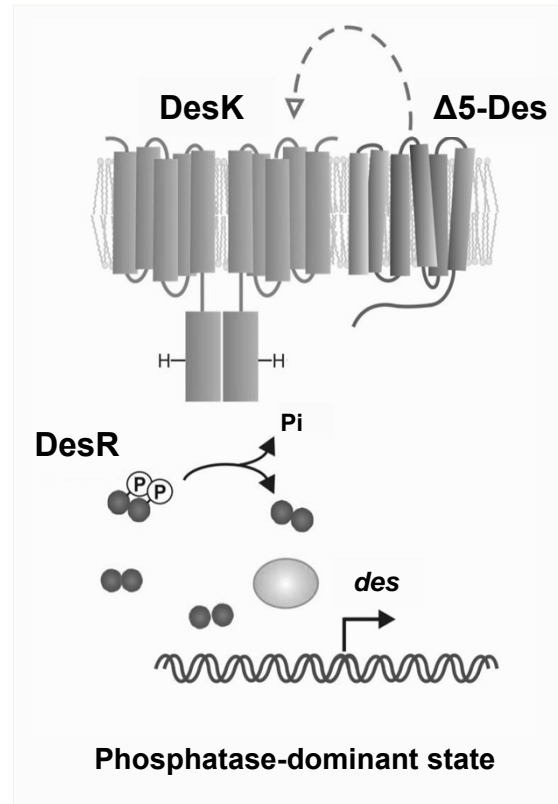

Supplement: Figure S1 — The Des signaling pathway for regulation of UFAs synthesis in Bacillus subtilis. DesK could assume different signaling states in response to changes in membrane fluidity. An increase in the order of the acyl chains of membrane lipids (less fluid membrane) promotes a kinase-dominant state of DesK, which autophosphorylates and transfers the phosphate group to DesR. DesK-mediated phosphorylation of DesR enables interaction of DesR-P with des promoter and RNA polymerase, resulting in transcriptional activation of des. Then Δ5-Des is synthesized and desaturates the acyl chains of membrane phospholipids. These newly synthesized UFAs cause a decrease in the order of membrane lipids (more fluid membrane) favoring a phosphatase-dominant state of DesK, leading to dephosphorylation of DesR and thus turning off des transcription. [file mbo30003-0213-sd1.pdf]

**A**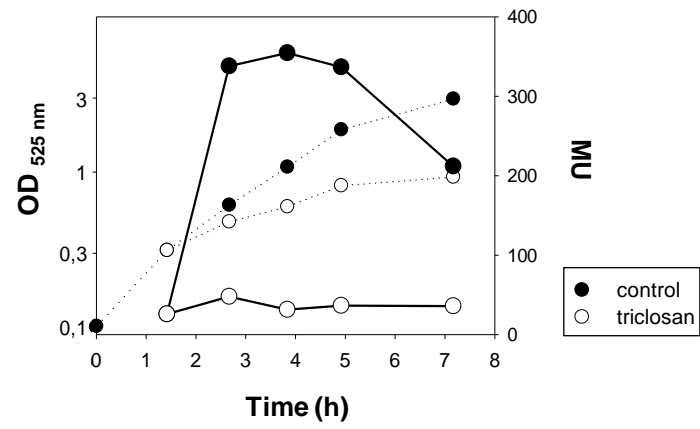**B**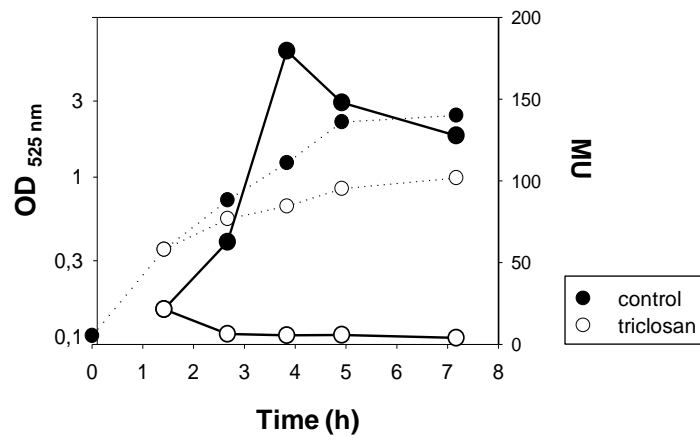

Supplement: Figure S2 — Effect of triclosan on des transcription in cerulenin-sensitive or cerulenin-resistant strains. Cells of B. subtillis AKP3 (fabF cerS, amyE::Pdes-lacZ) (A) or BLUP87 (fabF1 cerr, amyE::Pdes-lacZ) (B) were grown in LB medium at 37°C to an OD525 of 0.35 and then were treated with triclosan 0.4 µg mL −1 (white circles) or untreated (black circles). Cultures were further transferred to 25°C. β-galactosidase specific activities (in Miller units, MU) were determined at the indicated time intervals. Dotted lines: OD525, solid lines: β-galactosidase specific activities. Values are representative of three independent experiments). [file mbo30003-0213-sd2.pdf]
